# Editorial: New understandings and research in anal squamous cell carcinoma

**DOI:** 10.3389/fonc.2022.1131678

**Published:** 2023-01-16

**Authors:** Stefano Kim, Berardino De Bari, Laurie Spehner

**Affiliations:** ^1^ Clinical Investigational Center, INSERM CIC-1431, Centre Hospitalier Universitaire de Besançon, Besancon, France; ^2^ Department of Oncology, Sanatorio Allende, Cordoba, Argentina; ^3^ Service de radio-oncologie, Réseau Hospitalier Neuchâtelois (RHNE), La Chaux-de-Fonds, Switzerland; ^4^ INSERM U1098 Interactions Hôte-Greffon-Tumeur & Ingénierie Cellulaire et Génique, Besançon, France

**Keywords:** anal carcinoma, immunotherapy combined therapy, chemotherapy, biomarkers, researches

This is an exciting moment for researchers and clinicians involved in the treatment of Squamous Cell Carcinoma of the Anus (SCCA). Since the consolidation of the chemoradiotherapy with mitomycin and fluoropyrimidine as a standard treatment for localized disease, no clear advances have been achieved for several decades, and the disease remained out of the focus of the development of breakthrough strategies, partly due to its low incidence, especially at advanced stages. However, in last years, well-designed prospective academic and industrial trials are surging, as well as numerous biological and translational studies to better understand this orphanage cancer.

Recent data consolidated taxane-based doublet or triplet chemotherapies as standard treatments in front line for metastatic or non-resectable locally advanced diseases, demonstrated the interest of PD1/L1 inhibitors in a subgroup of patients in chemorefractory situation, and also validated the liquid biopsy for HPV oncoproteins (HPV ctDNA) as the best prognostic tool with high sensitivity and specificity in localized and advanced stages.

In this collection entitled “New understanding and researches in SCCA”, 8 articles enrich the present knowledge in SCCA and highlight the future directions in its treatment strategies. We believe that these publications will help clinicians in their daily practice, and give an insight in current status of research in SCCA.

Three ongoing prospective trials are presented. One in locally advanced stage, one in first-line metastatic disease, and one in chemorefractory patients.

In chemorefractory patients, several prospective trials evaluated PD-1/L1 inhibitors as monotherapy in advanced SCCA, refractory to at least one line of systemic treatment. Even though these trials confirmed the interest of immunotherapy, its efficacy is limited to around 15% of patients. VolaTIL trial (NCT) presented here is a phase II clinical trial aiming at improving the anti-PD1/PDL1 efficacy by using a therapeutic cancer vaccine composed of two peptides derived from telomerase (UCPVax), in HPV-related cancers including SCCA (Rebucci-Peixoto et al.). Telomerase is an enzyme that is overexpressed in more than 90% of human cancers, and thus prevent the tumor senescence conferring a form of immortality. Previous data have shown that anti-telomerase immunity of the patients was correlated to survival in different cancers, including SCCA (1). VolaTIL is a phase II study and its primary objective is the ORR of 45% at 4 months by iRECIST criteria, and its secondary objectives are OS, PFS, HRQoL, safety, the ability of UCPVax and atezolizumab to promote peripheral the activity of antigen specific T lymphocytes in the peripheral blood and to enhance the tumor cell infiltration by specific lymphocytes (Tumor Infiltrating Lymphocytes: TIL), the feasibility of TIL expansion in HPV+ cancer patients, and biomarkers. Forty-seven patients are expected to be included, and its final results are awaited during 2023.

In first-line metastatic or non-resectable locally advanced recurrent SCCA, taxane-based polychemotherapy settled a new threshold in the treatment standard. Epitopes-HPV02 study confirmed the high efficacy of mDCF (modified regimen of docetaxel, cisplatin, and 5-fluorouracil) (2), and InterAACT demonstrated a better tolerance of CP (carboplatin and paclitaxel) compared to CF (cisplatin and 5-fluorouracil) regimen (3). Recently, SCARCE-PRODIGE60 trial have failed to demonstrate a benefit of the addition of an anti-PDL1 to mDCF in a randomized phase II study, though it confirmed the high efficacy and tolerance of mDCF regimen as well as the feasibility of mDCF + immunotherapy (4). PODIUM 303/InterAACT 2 study presented here is a phase III international randomized double-blind study of retifanlimab (anti-PD1 inhibitor) or placebo plus carboplatin and paclitaxel (Rao et al.). Retifanlimab has already demonstrated its safety and efficacy in SCCA patients in PODIUM 202 phase II trial in chemorefractory patients (5). In PODIUM 303/InterAACT 2, approximately 300 patients with inoperable locally recurrent or metastatic SCCA not previously treated with systemic chemotherapy will be included. The randomization will be 1:1 and will be stratified by PD-L1 expression (<1% versus ≥1%), extent of disease (locally recurrent versus metastatic), and region. The primary study endpoint is PFS per RECIST v1.1 by BICR (HR of 0.67, with 83% of power and one-sided alfa error of 2.5%), and the key secondary endpoint is OS. Of note, crossover to open-label retifanlimab is allowed for patients assigned to placebo upon verification of disease progression by BICR. Additional secondary endpoints include overall response rate (ORR), duration of response (DOR), disease control rate (DCR) by BICR, safety, and retifanlimab pharmacokinetics. The trial is actually ongoing in 11 western countries, and could settle a new standard in case of positive results. At date, a similar phase III EA2176 trial (NCT04444921) is concurrently ongoing. This is an open label multicentric study limited to US evaluating the interest of the addition of nivolumab to carboplatin and paclitaxel. The primary end-point is PFS, and 205 patients are planned to be included.

In locally advanced disease at diagnosis, ACCORD03 trial have failed to demonstrate a benefit of an induction chemotherapy with cisplatin and 5-fluorouracil, and at date, these patients receive the same chemoradiotherapy protocol than a small SCCA disease despite its significantly higher recurrence rate of up to 50%. INTERACT-ION trial presented here is a pivotal, open-label, phase II study to evaluate a novel induction treatment with the highly effective mDCF regimen in combination with ezabenlimab (anti-PD1 inhibitor) in patients with stage III SCCA disease (N+ or T4 N0) (Kim et al.). This is a logical step considering the 100% of disease control rate in chemo-naïve advanced SCCA patients (29/115) treated with DCF regimen, with 90% of ORR including 55% of CRR, and almost 25% of disease-free survival at 5 years in favor of its effectiveness in micrometastases (6). Besides, anti-PD1 immunotherapies have demonstrated outstanding results in neoadjuvant setting in different tumors including squamous-cell head and neck carcinoma. Here, the authors propose a dynamic treatment algorithm based on clinical, pathological and biological response after 2 months of induction treatment. In case of a clinical objective response along with complete or near-complete pathological response at biopsy and a complete biological response at liquid biopsy (HPV ctDNA), patients will receive “involved node chemoradiotherapy” consisted of an irradiation of the primary tumor and only initially involved lymph node fields. The rational is to spare the non-involved regional lymph nodes to protect activated regional immunological memory cells, and thus maintain the anti-tumor immunity restored by mDCF plus immunotherapy. Then, these patients will continue with ezabenlimab in maintenance up to 10 months. The primary end-point is the CRR of more than 80% at 10 months from the treatment initiation, and main secondary endpoints include major pathological response and biological complete response after induction treatment, and after chemoradiotherapy. Other secondary endpoints are ORR, OS, PFS, RFS, HRQoL, and biomarkers

Squamous-cell carcinoma of the rectum (SCCR) is a very rare situation and its prognosis is poorer than SCCA and adenocarcinoma of the rectum (ACR). The 5-years OS rate is about 30% for a stage III disease (compared to 65% for SCCA and ACR) and less than 10% for a stage IV disease (compared to 20% for ACR and 30% for SCCA). In this collection, Hervé et al. describe 9 consecutive patients with advanced SCCR treated with 8 cycles of mDCF. All evaluable patients were positive for HPV. Six patients had a locally advanced disease (5 patients with T4 disease, and 4 patients with N+ disease), and 3 patients had synchronous or metachronous metastases. ORR was achieved in 87.5% and CRR was observed in 25% of patients after mDCF. The disease control rate was 100% during chemotherapy. One patient with metachronous liver metastasis underwent to liver surgery after mDCF and a pathological complete response was observed. Other 8 patients received a chemoradiotherapy of the primary tumor, and six of them (75%) presented a complete response, including a pathological complete response in the unique patient that underwent surgery of his primary tumor. After a median follow-up of 33 months, 78% of patients were alive and free of disease, and the median OS was not reached at 6 years. This article confirms the high efficacy of mDCF regimen observed in SCCA and should be considered as a standard in advanced SCCR. It also highlights the importance of complementary management with a majority of patients being alive at 6 years. This live-saving strategy is immediately applicable in the daily practice, and we believe that these patients should be treated in the referent centers by a multidisciplinary team.

Looking at the radiation oncology world, 2 retrospective studies are presented in this collection, one of them dealing with the role of modern irradiation techniques and with MRI-based radiomics in SCCA; and one exploring the role of potentially curative local chemoradiotherapy in metastatic SCCA patients.

Patterns of treatment for SCCA have changed over the time in the last decades. Indeed, we understood the detrimental impact of a longer overall treatment time, the explored different chemotherapy schedules, and we implemented newer and more conformal radiotherapy techniques.

In the study by Bonù et al. presented in this collection, the authors showed the positive impact of the modern RT techniques. They showed a reduction of the rate of severe acute toxicity with the implementation of Intensity Modulated Radiotherapy (IMRT), and an improvement of the local control, colostomy free survival and overall survival rates, probably related both to the optimization of the treatments schedules, but also to the introduction of the Image Guided Radiotherapy (IGRT), allowing a better targeting of the treatment volumes. These data confirm the positive impact of the IMRT already showed by the phase II RTOG 0529 trial published in 2013 (7). In this study, the authors compared their results with those obtained, in terms of toxicity, in the conventional radiation/5FU/MMC arm of the RTOG 9811 trial. They showed that the adoption of the IMRT delivered with a Simultaneous Integrated Boost technique, obtained a significant reduction in acute grade 2+ hematologic, 73% (vs 85%, P=.032), grade 3+ gastrointestinal, 21% (vs 36%, P=.0082), and grade 3+ dermatologic AEs 23% (49%, P<.0001).

Another Phase II study by Joseph et al. confirmed the low rates of acute toxicity in a prospective cohort of 57 patients treated with helical tomotherapy (8). Noteworthy, these prospective data have been confirmed by several retrospective series published before and after the publication of the results of the RTOG 0529 (9). Nevertheless, looking at NCCN guidelines, IMRT is still not considered the standard of care in the treatment of SCCA. Indeed, based on the low quality of the available data, the NCCN panel could only conclude “…that IMRT is preferred over 3D conformal RT”.

We consider that it is time to build a well-designed randomized multicentric phase III study, in order to definitively assess the superiority of modern therapeutic chemoradiotherapy approaches over the 3D conformal RT.

Radiomics and radiogenomics are being tested in clinical oncology, and it has been already showed that radiomics analyses could predict the clinical behavior of several gastrointestinal malignancies. The rarity of SCCA is a major limiting factor to realize such a kind of studies in this clinical setting. This is the case also for the data presented by Bonù et al. In a small proportion of the patients presented in their study, the authors showed that the most important feature in T2-weighted MR images to predict loco-regional recurrence is signal intensity, with a smaller contribution coming from LAHGLE and total volume. Interestingly, authors present hypothesis-generating data, with some radiomics features that could be related to tumor heterogeneity and behavior. A dose painting approach based on the radiomics features of the tumor is, in our opinion, a very interesting approach that could drive future studies on dose escalation (or de-escalation) in radiotherapy in SCCA patients.

The second article explored the role of local treatments as a consolidation therapy in advanced SCCA patients exposed to DCF polychemotherapy (Grave et al.). Here, 16 SCCA patients treated with DCF polychemotherapy in the context of 3 prospective trials were selected. All these patients achieved an objective response to chemotherapy and were treated by a chemoradiotherapy on their primary tumor site. After a median follow-up of 42 months, the authors showed, in this very selected group of patients, high rates of complete local response, comparable to those usually reported in non-metastatic patients. Noteworthy, these results were obtained despite interruptions due to toxicities and low compliance to concomitant chemotherapy (explained by the high rate of hematological toxicities that might have been fostered by the upfront CT). This is the first report, based on prospective data, exploring the role of pelvic chemoradiotherapy in metastatic SCCA patients.

The idea of delivering curative treatments on the primary tumors also in patients presenting a metastatic disease at diagnosis has been explored in several retrospective and prospective series. All the available studies confirmed the interest of such a kind of ambitious approaches in well selected patients. Concerning metastatic SCCA, Wang et al. published a retrospective study in 2019. The authors identified 437 patients treated with chemotherapy alone and 1,020 patients treated with pelvic chemoradiotherapy between 2004 and 2015 and compared outcomes. After a median follow-up of 17.3 months, univariate and multivariate analyses revealed that patients who received a local treatment presented a better overall survival (10). A study by Eng et al. on a smaller population of 77 SCCA patients confirmed that a multidisciplinary management including systemic chemotherapy followed by a local treatment with curative intent, including surgery and RT, improved OS up to 53 months (11).

Despite the methodological limits of these studies and the caution that should be used in the interpretation of the results, these studies sustain the multidisciplinary treatment of advanced SCCA diseases.

Whilst new combination therapies are being developed to improve the rate of complete remission and survival of patients with SCCA, there is still no validated predictive biomarker. Thus, the identification of easily analyzable biomarkers that can predict the efficacy of these treatments is an important issue for SCCA patients.

Integration of HPV genome into the host genome has been shown to be correlated with disease progression in cervical and anal cancer. However, episomal HPV DNA can also be detected in invasive carcinoma. Debernardi et al evaluated the prognostic value of HPV integration status and molecular profile in SCCA patients treated by DCF chemotherapies. To conduct these analyses, tumor samples from 39 patients included in Epitopes-HPV02 study were available. The authors explored the impact of 43 somatic mutations of oncogenes on SCCA patients’ survival, nonetheless, none were significantly correlated with progression free survival. Furthermore, they observed a majority of integrated HPV forms in these patients. Patients with an integrated HPV form had lower progression free survival compared to patients with an episomal HPV DNA. These data were not significant and further investigations should be undertaken to confirm this observation. Nevertheless, the better progression free survival observed in patients harboring episomal form raises the hypothesis of a predictive impact of HPV integration status on treatment efficacy in advanced SCCA patients.

PD-L1 expression has been shown to be prognostic in many cancers and used in consideration of checkpoint inhibitor immunotherapy. Nevertheless, the threshold of PD-L1 expression positivity currently used in SCCA patients may not be optimal to select patients who could benefit from the immunotherapy. This hypothesis was studied by Chan et al. who evaluated the prognostic value of PD-L1 and CD8 expression in SCCA patients treated by chemoradiotherapy (Chan et al.). For this, they measured the expression of these 2 markers by fluorescence immunohistochemistry in 63 SSCA patients whose tumor samples were available. Different thresholds were studied, and the data showed that a threshold for tumor PD-L1 expression of ≥5% was associated with better overall survival, which is not the case with the currently used threshold of 1%. If the CD8 expression did not influence the overall survival, the combination of these 2 markers showed that overall survival was driven by tumor PD-L1 expression but may be influenced by CD8 infiltration levels. The authors showed that a threshold for PD-L1 expression (tumor PD-L1 ≥5%) was a prognostic biomarker associated with overall survival in SCCA patients treated by chemoradiotherapy. While the prognostic value of this new threshold needs to be validated in future trials, these data suggest that tumor PD-L1 ≥5% may predict responses to immunotherapies in SCCA patients.

These results demonstrate the interest of identifying and validating the SCCA-related predictive biomarkers. These biomarkers will allow a better stratification of SCCA patients and will limit the risks of recurrence by adapting treatments in these patients.

## Author contributions

All authors listed have made a substantial, direct, and intellectual contribution to the work and approved it for publication.
